# Evaluation of ^68^Ga-Labeled MG7 Antibody: A Targeted Probe for PET/CT Imaging of Gastric Cancer

**DOI:** 10.1038/srep08626

**Published:** 2015-03-03

**Authors:** Bing Xu, Xiaowei Li, Jipeng Yin, Cong Liang, Lijuan Liu, Zhaoyan Qiu, Liping Yao, Yongzhan Nie, Jing Wang, Kaichun Wu

**Affiliations:** 1 State Key Laboratory of Cancer Biology & Institute of Digestive Diseases, Xijing Hospital, The Fourth Military Medical University, Xi'an, China; 2 Department of Nuclear Medicine, Xijing Hospital, The Fourth Military Medical University, Xi'an, China

## Abstract

MG7-Ag, a specific gastric cancer-associated antigen, can be used to non-invasively monitor gastric cancer by molecular imaging with positron emission tomography/computed tomography (PET/CT). In this study, we prepared and evaluated a ^68^Ga-labeled MG7 antibody as a molecular probe for nanoPET/CT imaging of gastric cancer in a BGC-823 tumor xenografted mouse model. Macrocyclic chelator 1,4,7-triazacyclononane-N,N0,N00-triacetic acid (NOTA)-conjugated MG7 antibody was synthesized and radiolabeled with ^68^Ga (t_1/2_ = 67.71 min). Then, ^68^Ga-NOTA-MG7 was tested using in vitro cytological studies, in vivo nanoPET/CT and Cerenkov imaging studies as well as ex vivo biodistribution and histology studies. The in vitro experiments demonstrated that ^68^Ga-NOTA-MG7 has an excellent radiolabeling efficiency of approximately 99% without purification, and it is stable in serum after 120 min of incubation. Cell uptake and retention studies confirmed that ^68^Ga-NOTA-MG7 has good binding affinity and tumor cell retention. For the nanoPET imaging study, the predominant uptake of ^68^Ga-NOTA-MG7 was visualized in tumor, liver and kidneys. The tumor uptake reached at its peak (2.53 ± 0.28%ID/g) at 60 min pi. Cherenkov imaging also confirmed the specificity of tumor uptake. Moreover, the biodistribution results were consistent with the quantification data of nanoPET/CT imaging. Histologic analysis also demonstrated specific staining of BGC-823 tumor cell lines.

The emergence of molecular imaging has been a milestone in the development of radiographics in the early twenty-first century. Molecular imaging has led to substantial advances in the diagnosis of diseases, especially in the field of cancer. Molecular imaging makes it possible to directly, dynamically, and non-invasively monitor the pathological processes of cancer in real-time at the cellular and molecular levels^1^,^2^. Unlike traditional anatomical imaging methods, the following three essential factors must be considered in molecular imaging: 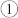 suitable molecular imaging probes, 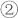 biological signal amplification systems, and 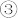 highly sensitive imaging apparatus^2^. Generally, the development of a suitable molecular imaging probe is the most important of these factors. Molecular probes are compounds that combine the targeted ligands (such as peptides and antibodies) and substances to produce imaging signals^3^,^4^,^5^. Numerous molecules associated with the development of cancer have been discovered in recent years, making targeted molecular imaging possible.

Gastric cancer, with its high incidence and mortality, rapid progression and deterioration, has developed into a serious health problem, particularly in China^6^,^7^,^8^. Thus, an effective method for diagnosing gastric cancer at an early stage is urgently needed. MG7-Ag, a specific gastric cancer-associated antigen identified by D Fan et al^9^, is distinguished only in the presence of gastric cancer lesions. MG7-Ag is expressed in 91.2% of gastric cancer lesions and in 0.0% of the normal gastric mucosa^10^,^11^. Gastric monoclonal antibody MG7 was primary obtained by immunizing BALB/C mice with the poorly differentiated adenocarcinoma gastric cancer cell line MKN-46-9. Immunohistochemistry and immunofluorescence confirmed the targeting activity of the MG7 antibody^11^. Given that the MG7 antibody might be of great value in diagnosing gastric cancer, we took advantage of this antibody as a targeting molecule in developing a non-invasive probe that could be used to visually diagnose gastric cancer in vivo. Considering that a variety of ligands can be radiolabeled, nuclear modalities, such as single photon emission computed tomography (SPECT) and positron emission tomography (PET), are ideally suited for imaging molecular events. PET, a world-renowned, groundbreaking, high-tech imaging modality, has superior sensitivity in the early diagnosis of cancer and other diseases^12^,^13^,^14^. More importantly, the development of positron emission tomography/computed tomography (PET/CT) integration imaging makes PET a more powerful apparatus for demonstrating detailed molecular information of the function and metabolism by CT scans, providing precise anatomical localization of lesions. PET/CT wins the advantages of the two modalities and creates spectacular high resolution images that combine anatomical and functional information simultaneously^15^. ^18^F-FDG, the most commonly used clinical PET radiotracer, has substantially improved tumor diagnosis, but it is far from perfect^16^,^17^,^18^ given its high cost, lack of specificity and cyclotron dependence^19^. Fortunately, ^68^Ga has a reasonable half-life (67.71 min) and favorable positron emission (89%)^20^,^21^. Typically, ^68^Ga connects with targeting molecules through a bifunctional chelating agent^22^,^23^,^24^. The macrocyclic chelator 1,4,7-triazacyclononane-N,N0,N00-triacetic acid (NOTA) has been reported to form an extremely stable pattern when it interacts with ^68^Ga, and the reaction can be performed under mild conditions to ensure the biological activity of targeting molecules^25^. In this research, NOTA was selected as a chelator, and a MG7 analog, NOTA-conjugated MG7 antibody, was synthesized and radiolabeled with the positron emitter ^68^Ga. The in vitro stability, partition coefficient, tumor cell line characterization, tumor cell uptake and retention of ^68^Ga-NOTA-MG7 were investigated. The feasibility of ^68^Ga-NOTA-MG7 to image gastric cancer tissues using nanoPET/CT and Cerenkov imaging was further evaluated in a BGC-823 tumor xenograft nude mouse model.

## Results

### Radiochemistry, log P value and in vitro stability

**Figure 1:**
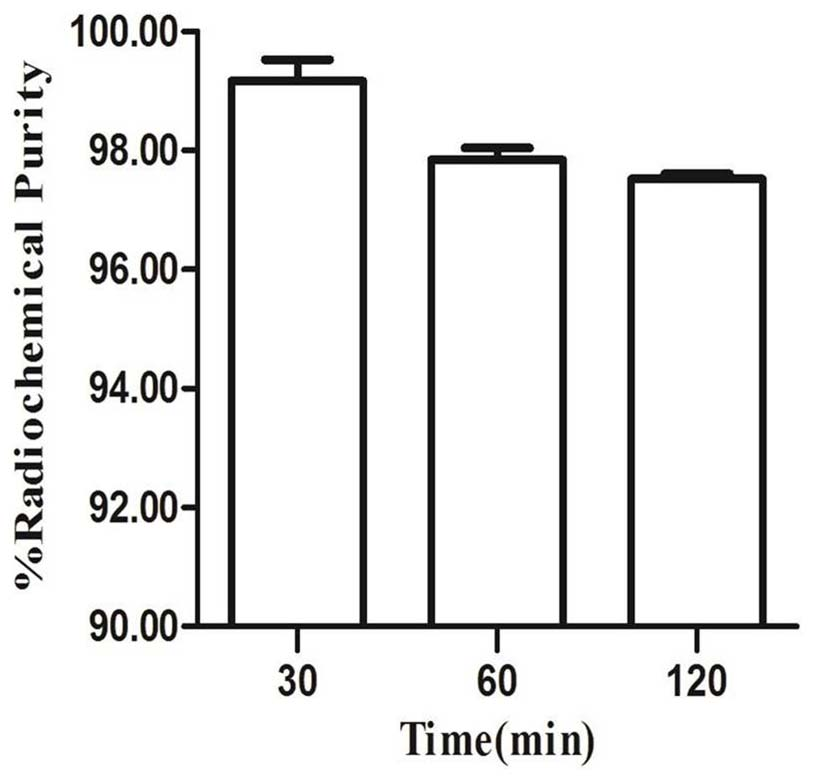
Stability of ^68^Ga-NOTA-MG7 in 50% fetal bovine serum at 37°C for 30, 60 and 120 min (n = 3, mean ± SD).

The radiolabeling efficiency of ^68^Ga-NOTA-MG7 was evaluated using a radio-thin-layer chromatography (TLC) method and the radiolabeling efficiency was approximately 99% without purification, while the free ^68^Ga^3+^ remained at the origin of the TLC plate. The octanol-water partition coefficient (log P) of ^68^Ga-NOTA-MG7 was −2.42 ± 0.11, indicating that ^68^Ga-NOTA-MG7 was highly hydrophilic. The in vitro stability of ^68^Ga-NOTA-MG7 was analyzed in 50% fetal bovine serum with different time intervals (30, 60 and 120 min) at 37°C. The stability is presented as the percentage of intact ^68^Ga-NOTA-MG7 according to radio-TLC analysis and the result is shown in Fig. 1. After 120 min of incubation, more than 97% of ^68^Ga-NOTA-MG7 remained intact, indicating it has excellent stability.

### Cell Immunofluorescence

**Figure 2:**
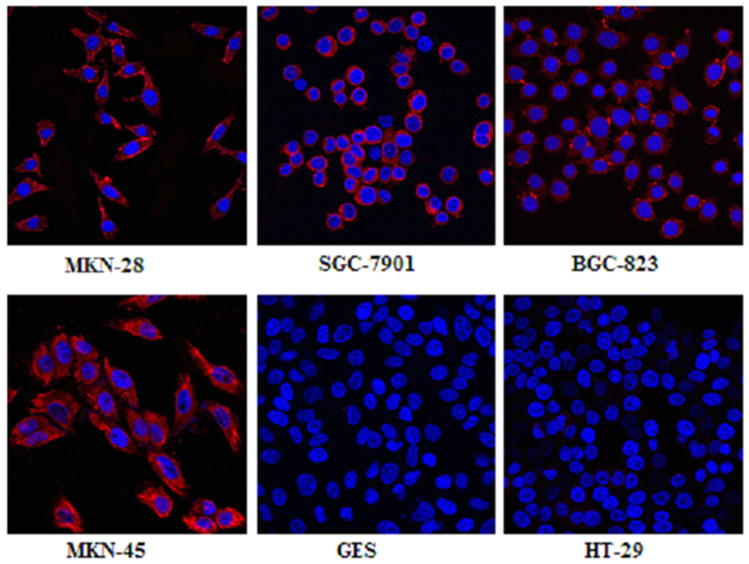
Laser scanning confocal microscopy imaging of different cell lines (MKN-28, SGC-7901, BGC-823, MKN-45, and GES, HT-29) after incubation with MG7 antibody showed that the expression of MG7-Ag was in the cell membrane and cytoplasm in each of gastric cell line, and there was little MG7-Ag expression in the normal gastric epithelial cell lines (GES) and colon cancer cell lines (HT-29). All images were acquired under the same condition and displayed at the same scale. Magnification: 60×.

Cell immunofluorescence studies were performed in gastric cancer cell lines, normal gastric epithelial cell lines and colon cancer cell lines. The expression of MG7-Ag was confirmed in the cell membrane and cytoplasm in each gastric cell lines. There was little expression of MG7-Ag in GES(normal gastric epithelial cell lines) and HT-29(colon cancer cell lines) (Fig. 2).

### Cell Uptake and Efflux

**Figure 3:**
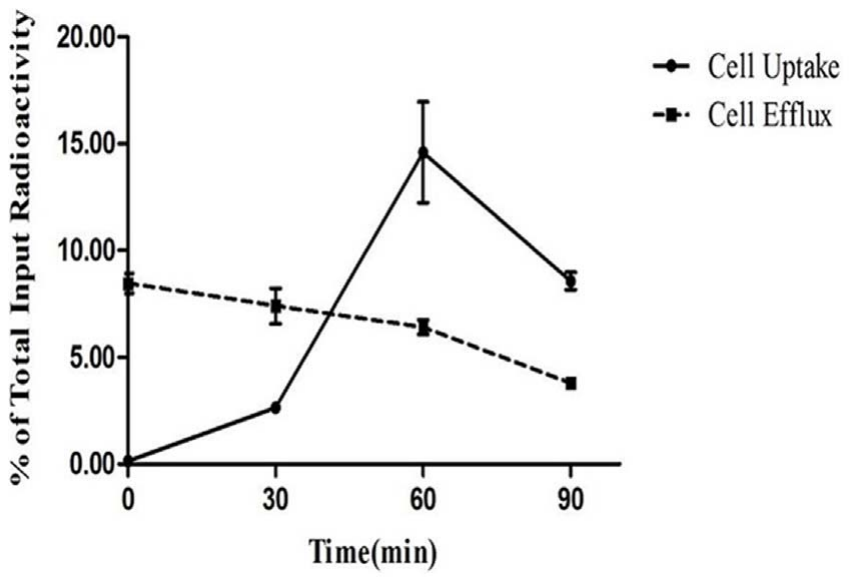
Cell uptake and efflux studies of ^68^Ga-NOTA-MG7 in BGC-823 cells (n = 6, mean ± SD). The background readings are reflected at time 0.

Cell uptake and cellular retention of ^68^Ga-NOTA-MG7 were examined in BGC-823 tumor cells. The cell uptake study confirmed that ^68^Ga-NOTA-MG7 binds to BGC-823 tumor cells. After 30 min of incubation, approximately 2.64% of ^68^Ga-NOTA-MG7 uptake in BGC-823 cells was detected. After 60 min of incubation, the uptake of ^68^Ga-NOTA-MG7 reached a maximum of 14.60% of the total input radioactivity (Fig. 3, solid line). The cell efflux study showed that ^68^Ga-NOTA-MG7 had relatively good cell retention in BGC-823 cells during the 60 min of cell efflux study, and approximately 2.15% (from 8.57% to 6.42% of total input radioactivity) of ^68^Ga-NOTA-MG7 efflux was detected. However, after 60 min, the cell efflux intensified, and approximately 4.78% (from 8.57% to 3.79% of the total input radioactivity) of ^68^Ga-NOTA-MG7 efflux was detected (Fig. 3, dotted line).

### Nano PET/CT imaging

**Figure 4:**
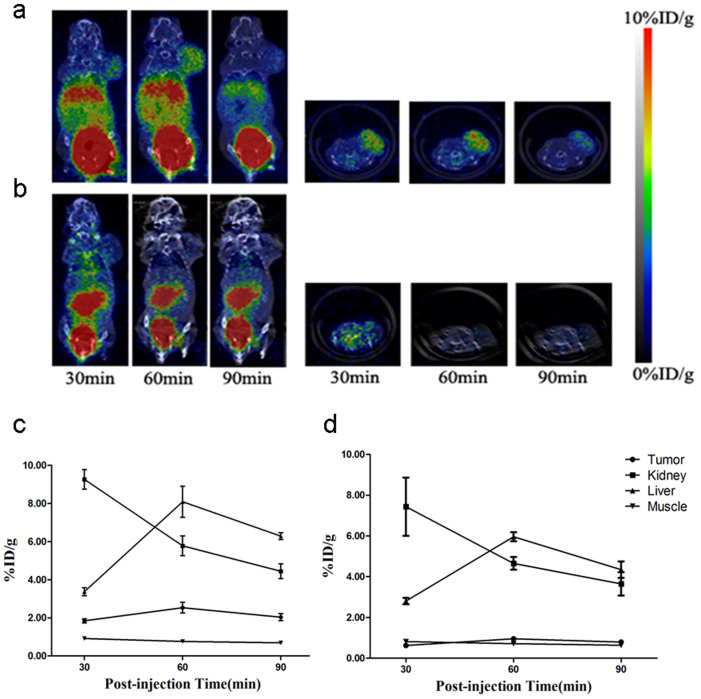
NanoPET/CT study of subcutaneous BGC-823 tumor-bearing nude mice after intravenous (i.v.) injection of 7.4 MBq of ^68^Ga-NOTA-MG7. (a). Decay-corrected whole-body nanoPET/CT images of nude mice bearing BGC-823 tumor at 30, 60 and 90 min pi. (b). Blocking experiment with co-injection of 20 mg/kg of MG7 antibody at 30, 60 and 90 min pi. (c). Time–activity curves (TACs) of the tumor, kidney, liver and muscle after intravenous (i.v.) injection of ^68^Ga-NOTA-MG7. (d). Time–activity curves (TAC) of the tumor, kidney, liver and muscle after co-injection with MG7 antibody (20 mg/kg of mouse body weight). ROIs are shown as the mean%ID/g ± SD (n = 3/group).

The tumor-targeting efficacy and pharmacokinetic pattern of ^68^Ga-NOTA-MG7 were evaluated in nude mice bearing BGC-823 gastric cancer xenograft tumors (n = 3) at multiple time points (30, 60 and 90 min) with static scans (Fig. 4a). The uptake of tumor and major organs were quantified based on ROI analysis of the nanoPET images (Fig. 4c). The tumor was clearly visible with high contrast relative to background within 30 min (1.84 ± 0.11%ID/g), and the signal peaked at 60 min (2.53 ± 0.28%ID/g) pi. Predominant uptake of ^68^Ga-NOTA-MG7 was also visualized in the liver and kidneys, and as a function of time, the probe was excreted. The kidney uptake values were calculated (9.26 ± 0.52%ID/g, 5.78 ± 0.52%ID/g, and 4.44 ± 0.39%ID/g) at 30, 60 and 90 min pi, respectively. The liver uptake values were calculated (3.37 ± 0.21%ID/g, 8.09 ± 0.81%ID/g, and 6.28 ± 0.19%ID/g) at 30, 60 and 90 min pi, respectively. The radioactivity uptake of the probe in other organs was relatively low.

### Blocking Experiment

The target specificity of ^68^Ga-NOTA-MG7 was achieved with a blocking experiment where the probe was co-injected with MG7 antibody (20 mg/kg of mouse body weight) (Fig. 4b). The tumor uptake was significantly reduced and the uptake values were calculated (0.62 ± 0.04%ID/g, 0.95 ± 0.11%ID/g, and 0.79 ± 0.03%ID/g) at 30, 60 and 90 min pi, respectively. The presence of non-radiolabeled MG7 antibody also decreased with the uptake of ^68^Ga-NOTA-MG7 in the liver and kidney (Fig. 4d).

### Cerenkov imaging

**Figure 5:**
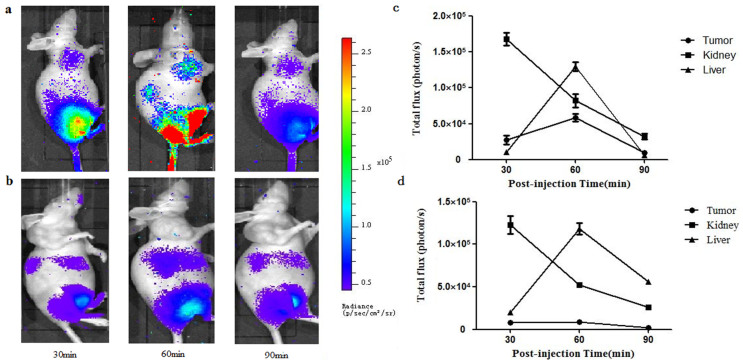
(a). Cherenkov imaging of subcutaneous BGC-823 tumor-bearing nude mice at 30, 60 and 90 min after intravenous (i.v.) injection of 18.5 MBq of ^68^Ga-NOTA-MG7. (b). Blocking experiment with co-injection of 20 mg/kg of MG7 antibody at 30, 60 and 90 min pi. (c). Dynamic changes of Cerenkov signal in the tumor, kidney and liver after intravenous (i.v.) injection of ^68^Ga-NOTA-MG7. (d). Dynamic changes of Cerenkov signal in the tumor, kidney and liver after co-injected with MG7 antibody (20 mg/kg of mouse body weight). All images were acquired under the same conditions and displayed on the same scale.

Cerenkov imaging was used to evaluate the tumor-targeting efficacy of ^68^Ga-NOTA-MG7 at different time points (30, 60 and 90 min). The tumor uptake reached its plateau at approximately 60 min pi, which was consistent with the PET/CT results (Fig. 5a). The tumor uptake was significantly reduced when the probe was co-injected with MG7 antibody (20 mg/kg of mouse body weight) (Fig. 5b). The uptake in the tumor and major organs was quantified based on ROI analysis of the Cerenkov images. High Cerenkov signal was observed in the tumor, liver and kidney (Fig. 5c, 5d).

### Biodistribution study

**Figure 6:**
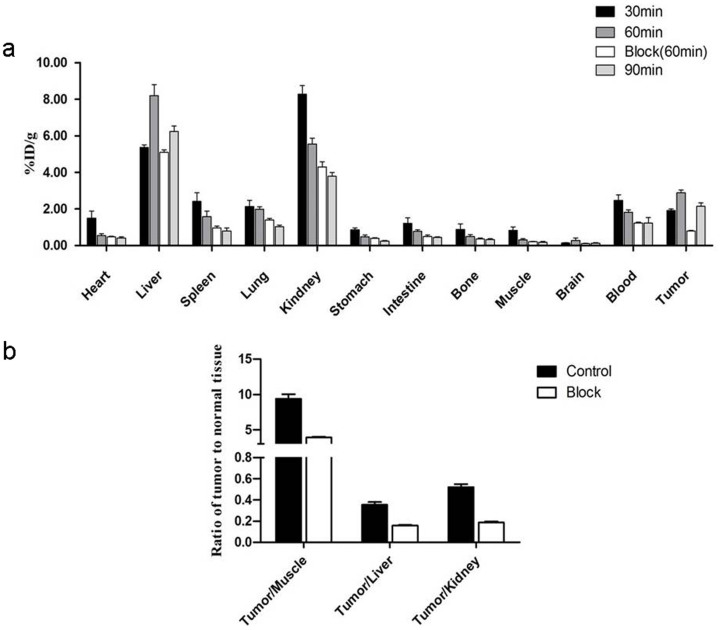
(a). Biodistribution of ^68^Ga-NOTA-MG7 in nude mice bearing BGC-823 tumors at 30, 60 and 90 min and blocking experiment with co-injection of 20 mg/kg of MG7 antibody at 60 min (n = 3/group). (b). Ratio of the tumor-to-major organs (muscle, liver and kidney) based on the biodistribution data at 60 min. The error bar was calculated as the standard deviation (n = 3/group).

The biodistribution study was performed in BGC-823 tumor xenograft models. High levels of ^68^Ga-NOTA-MG7 uptake were detected in the tumor, liver and kidneys. Tumor uptake increased as a function of time from 1.91 ± 0.08%ID/g at 30 min to 2.72 ± 0.40%ID/g at 60 min pi, whereas the presence of unlabeled MG7 antibody significantly reduced the tumor uptake to 0.80 ± 0.03%ID/g (P < 0.01) in the blocking group at 60 min. The results were consistent with the quantification data of nanoPET/CT imaging (Fig. 6a). Furthermore, for the non-blocking group, the uptake ratios of tumor to muscle, liver and kidney at 60 min pi were 9.74 ± 1.69, 0.34 ± 0.07, and 0.49 ± 0.09, respectively, while the corresponding values for the blocking group were 4.93 ± 0.62, 0.20 ± 0.01, and 0.24 ± 0.01, respectively (Fig. 6b).

### Histology

**Figure 7:**
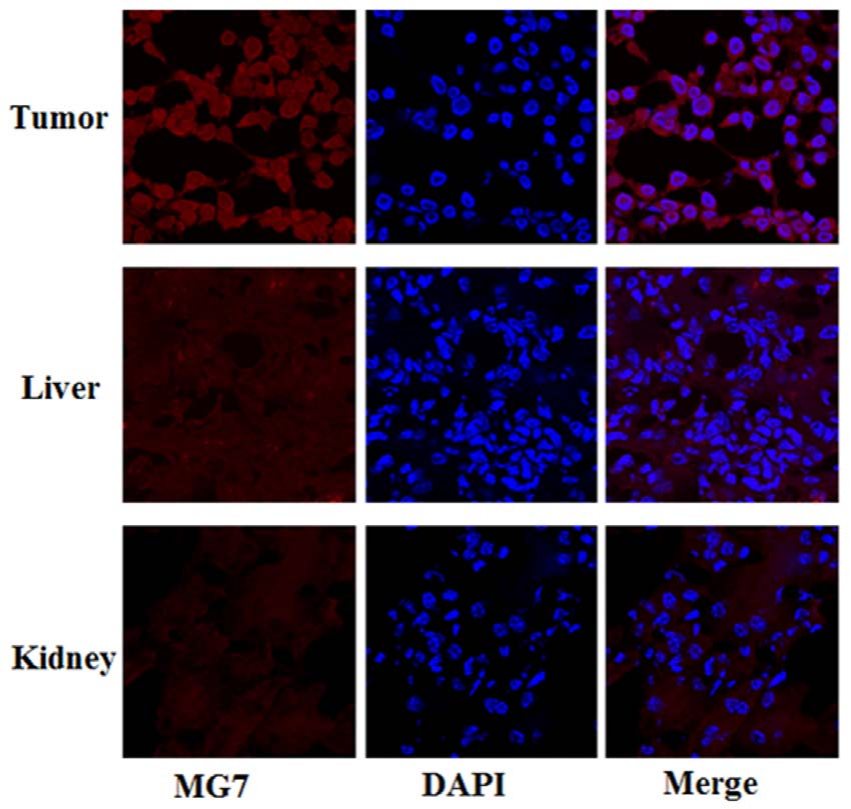
Immunofluorescence of the tumor, liver and kidney tissue sections. The tissue slices were incubated with TRITC for 30 minutes. DAPI was used to achieve nuclear staining. The results demonstrated that ^68^Ga-NOTA-MG7 was specifically bonded to the BGC-823 tumor cell lines. The staining of the mouse liver and kidney both revealed relatively low signals, indicating that these tissues do not express significant MG7-Ag. All images were acquired under the same conditions and displayed at the same scale. Magnification: 120×.

The ex vivo tumor-targeting efficacy and pharmacokinetic pattern of ^68^Ga-NOTA-MG7 were also evaluated in nude mice bearing BGC-823 gastric cancer xenograft tumors (n = 3). Sixty minutes after injection, the tumor, liver and kidneys were separated to perform immunofluorescence staining of frozen slices. The results also demonstrated the specific staining of BGC-823 tumor cell lines. The staining of the mouse liver and kidney had relatively low signals, indicating that these tissues did not express significant MG7-Ag. Therefore, uptake of ^68^Ga-NOTA-MG7 in the liver and kidney was largely unrelated to MG7 binding and more likely attributed to clearance of the tracer (Fig. 7).

## Discussion

In this study, we have successfully developed a targeted probe for PET/CT imaging of gastric cancer. With the combination of ^68^Ga-labeled MG7 antibody probe and PET/CT, we confirmed that molecular imaging of gastric cancer can be achieved with PET/CT imaging. Many previous studies in our laboratory have demonstrated the specificity of the MG7 antibody in gastric cancer^11^,^26^,^27^,^28^,^29^. To achieve nanoPET/CT targeted imaging of the MG7 antibody, NOTA was selected as a bridge that connected the antibody and ^68^Ga. NOTA is a superior alternative bifunctional chelator because it allows for highly efficient radiolabeling at room temperature. As a result, the biological activity of MG7 antibody cannot be affected. In addition, chelating with ^68^Ga forms a very stable pattern^25^,^30^. The radiolabeling efficiency, partition coefficient and stability of ^68^Ga-NOTA-MG7 were investigated and we confirmed that the probe has an extremely high labeling efficiency with optimistic aqueous solubility and stability. The in vitro cell immunofluorescence experiment showed strong evidence of specific MG7 antibody expression in gastric cancer cell lines. Utilization of BGC-823 cells for the cell uptake and efflux study demonstrated that ^68^Ga-NOTA-MG7 binds to BGC-823 gastric cells; even 90 min after incubation, there is still 8.57% of the total input radioactivity can be detected. A significant increase in ^68^Ga-NOTA-MG7 uptake was observed from 30 min, and the maximum uptake value was at 60 min. Furthermore, a cell retention study showed that ^68^Ga-NOTA-MG7 is slowly washed out from cells between 30 and 60 min, but the cellular efflux was significantly faster from the beginning of 60 min. In spite of this, 3.79% of the total input radioactivity can be detected after 90 min of incubation. These in vitro results are in agreement with further evaluation in animal models. Nano PET/CT imaging of ^68^Ga-NOTA-MG7 in nude mice bearing BGC-823 tumors at 30, 60 and 90 min after injection showed a high tumor-to-background. Predominant uptake of ^68^Ga-NOTA-MG7 was visualized in the tumor, liver and kidney as a function of time. The target specificity of ^68^Ga-NOTA-MG7 was demonstrated with a blocking experiment where the tracer was co-injected with non-radiolabeled MG7 antibody. The tumor uptake was significantly reduced. The uptake in the liver and kidneys was lower than the non-blocking group. The results from ex vivo biodistribution studies are consistent with the findings obtained from nanoPET imaging. The immunofluorescence staining of frozen sections of the tumor, liver and kidneys were performed immediately after the injection of ^68^Ga-NOTA-MG7 (for 60 min) to further evaluate the targeted specificity. Specific binding can be observed in the tumor cells, while there was no specific uptake in the liver and kidney cells. However, the probe accumulated in the interstitium of the liver and kidney slices. One possible explanation for this phenomenon is that it is evidence that the probe is metabolized through the liver and kidney. Cerenkov imaging makes it possible to translate the radionuclide imaging into optical Imaging, and the results are optimistically consistent with nanoPET/CT imaging. Several studies have reported on radiolabeling antibodies with ^68^Ga. However, most of them used a pre-targeted approach^31^,^32^,^33^. In this study, we demonstrated that a direct labeling method could also allow for targeted imaging. However, in spite of the great enthusiasm and developments in this area, the FDA has only approved three monoclonal antibodies for human use^34^.

## Conclusion

The radiosynthesis of ^68^Ga-NOTA-MG7 was achieved at a high yield under mild conditions. ^68^Ga-NOTA-MG7 exhibits optimistic stability as well as excellent cell uptake, internalization, and retention in BGC-823 gastric cancer cells. NanoPET/CT imaging and biodistribution studies demonstrated excellent tumor-targeting efficacy and specificity of ^68^Ga-NOTA-MG7 in xenograft mice models. ^68^Ga-NOTA-MG7 is a promising radiotracer for imaging gastric cancer in living subjects.

## Methods

### Materials

All commercially accessible chemicals (biochemical grade) were purchased from Sigma Aldrich (St. Louis, MO, USA) and without further purification. The NOTA-NHS-ester was purchased from Macrocyclis (Dallax, TX, USA). ^68^Ga was obtained from a ^68^Ge/^68^Ga generator (Isotope Technologies Garching GmbH, Garching, Germany). The mouse monoclonal antibody MG7 was produced and purified in State Key Laboratory of Cancer Biology, Institute of Digestive Diseases, Xijing Hospital, Fourth Military Medical University, PRC. NOTA-MG7 was purified and concentrated using an Amicon® Ultra-2 Centrifugal Filter Device (Merck KGaA, Darmstadt, Germany), and it was quantified using a BCA protein assay kit (Pierce, Rockford, IL, USA). The immunofluorescence was performed using laser confocal scanner (Olimpus, Fluoview FV10i, Tokyo, Japan). Radiochemical analysis was performed using a radioactive thin-layer chromatography scanner (radio-TLC) (Bioscan, Fairfield, CT, USA) to determine the radiochemical purities (RCPs) of ^68^Ga-NOTA-MG7. Radioactivity was measured using a dose calibrator (Biodex Medical Systems, Shirley, NY, USA) and the tissue radioactivity was counted using an automated gamma counter (Rihuan, Shanghai, China). The in vivo imaging was performed by nanoPET/CT (Mediso, Budapest, Hungary) and the IVIS Lumina II (Caliper, MA, Hopkinton).

### Synthesis of NOTA-MG7

MG7 antibody (1 mg, 6.67 nmol) was dissolved in ultrapure water (1 ml), and NOTA-NHS-ester (13.2 μg, 20 nmol) was added. The mixture underwent oscillation overnight at 4°C. Then, the mixture was purified using a Amicon® Ultra-2 Centrifugal Filter Device (cutoff: 30 KDa) and lyophilized for further applications. NOTA-MG7 was quantified with a BCA protein assay kit.

### Preparation of ^68^Ga- NOTA-MG7

^68^GaCl_3_ (0.74 GBq) was eluted from a ^68^Ge/^68^Ga generator using 0.05 M HCl. NOTA-MG7 (100 μg) was dissolved in water (1 μg/μl), and 200 μl of ^68^GaCl_3_ (55–74 MBq) was added. Finally, we adjusted the pH to 3.7 using sodium acetate (1.25 M). Then, the reaction mixture was incubated at room temperature for 30 min. The radiolabeling efficiency was measured by radio-thin-layer chromatography (TLC) using a 1:1 mixture of 10% ammonium acetate (aq.) and methanol as the developing solvent system. The product was sterilized through a 0.22-μm Millipore filter for later use.

### Partition Coefficient

To configure the octanol-water saturated solution, approximately 5 μCi (185 KBq) of ^68^Ga- NOTA-MG7 was added. The mixture was vigorously vortexed for at least 10 minutes before centrifugation; then, 200 μl of each layer was pipetted into a test tube, and the radioactivity was measured using a gamma counter. The partition value was formed as log P. The mean value was calculated from triplicate experiments.

### Stability

^68^Ga-NOTA-MG7 (5 μCi, 185 KBq) was added to 50% fetal bovine serum and incubated at 37°C for 30, 60 and 120 min. The radiolabeling efficiency was measured at specified time points by radio-thin-layer chromatography (TLC). The TLC findings were further confirmed by ultrafiltration using an Amicon® Ultra-2 centrifugal filter device.

### Tumor Cell Line Characterization

The different differentiation gastric cancer cell lines MKN-28 (well-differentiated), SGC-7901 (moderately differentiated), MKN-45 (poorly differentiated) and BGC-823 (poorly differentiated) were cultured in RPMI-1640 medium (Gibco®) supplemented with 10% fetal bovine serum at 37°C containing 5% CO_2_.

### Cell Immunofluorescence

The gastric cancer cell lines (MKN-28, SGC-7901, MKN-45, and BGC-823) were cultured in chamber slides until a sufficient number of cells were available. Cells were fixed in 4% paraformaldehyde for ten minutes and blocked in normal rabbit serum blocking solution for one hour; then, they were incubated with MG7 antibody, NOTA-MG7, at 4°C overnight, respectively. The next day, cells were washed three times with PBS and incubated with fluorescent secondary antibodies (tetramethylrhodamineisothiocyanate, TRITC) for 30 minutes. Nuclear staining was achieved using 4′,6-diamidino-2-phenylindole (DAPI). Normal gastric epithelial cell lines (GES) and colon cancer cell lines (HT-29) were selected as negative controls. Images were acquired using a laser confocal scanning microscope.

### Cell uptake and efflux

The gastric cancer cell line BGC-823 was selected to perform these studies. For cell uptake, cells were seeded into 24-well plates at a density of 1*105 cells per well 24 h prior to the experiment. The cells were then incubated with ^68^Ga-NOTA-MG7 (185 KBq/well) at 37°C for 30, 60 and 90 min. After incubation, the cells were washed three times with cold PBS and harvested with NaOH (0.1 M); then, the cell lysate was collected and measured by a gamma counter. For the efflux study, ^68^Ga-NOTA-MG7 (185 kBq/well) was incubated for 90 min at 37°C. Then, it was washed three times with PBS and incubated with cell culture medium for 30, 60 and 90 min. After being washed three times with PBS, cells were harvested by NaOH (0.1 M). Cell lysate was collected, and the remaining radioactivity was measured in a gamma counter. Experiments were performed two times with triplicate wells. The cell uptake and efflux data are presented as the percentages of the total input radioactivity after decay correction.

### Tumor-bearing murinemarine model

To further estimate the feasibility of intravital molecular imaging of ^68^Ga-NOTA-MG7, the gastric cancer xenograft model was established with subcutaneous injection of 5*10^6^ BGC-823 tumor cells into the front flank of female athymic nude mice. Approximately 2 to 4 weeks after inoculation, the tumor size was approximately 200 to 500 mm^3^ in volume by caliper measurements of the perpendicular dimensions. All animal experiments were approved by the local committee of Animal Care and Use, which are in accordance with the NIH guidelines.

### Nano PET/CT imaging

NanoPET/CT scans were performed using a rodent scanner. ^68^Ga-NOTA-MG7 (7.4 MBq/200 μl) was injected into each mouse under isoflurane anesthesia through tail vein. A ten-minute static scan was acquired at 30, 60 and 90 min. The images were reconstructed by a two-dimensional ordered-subsets exception maximum algorithm. For each nanoPET/CT scan, regions of interest (ROIs) were drawn over the tumor, muscle, liver and kidney on the decay-corrected whole-body coronal images. Time activity curves (TAC) were derived and visualized for each ROI. The target specificity of ^68^Ga-NOTA-MG7 was achieved by a blocking experiment wherein the probe was co-injected with MG7 antibody (20 mg/kg of mouse body weight).

### Cherenkov imaging

An IVIS Lumina II spectrum imaging system was used to record the images (exposure time: 60 s). Approximately 18.5 MBq of ^68^Ga-NOTA-MG7 was injected (via tail vein) into each mouse under isoflurane anesthesia. The images were acquired at 30, 60 and 90 min. For each scan, regions of interest (ROIs) were drawn over the tumor, liver and kidney. The blocking experiments were performed by co-injection with MG7 antibody (20 mg/kg of mouse body weight). The quantification was based on photon radiance (photon per second per square centimeter per steradian).

### Biodistribution Studies

The BGC-823 tumor bearing nude mice were injected with ^68^Ga-NOTA-MG7 (7.4 MBq/200 μl), respectively. Mice (n = 3) were sacrificed at 30, 60 and 90 min post-injection (pi), and the tissues of interest (heart, liver, spleen, lung, kidney, stomach, intestinal, bone, muscle, brain, blood and tumor) were separated immediately and weighed wet. The blocking experiment was performed by co-injection with MG7 antibody (20 mg/kg of mouse body weight) at 60 min pi. The radioactivity in the tissue was measured using a gamma counter. The data were presented as the percentage injected dose per gram of tissue (%ID/g).

### Histology

The nude mice bearing BGC-823 gastric cancer xenograft tumors (n = 3) were sacrificed at 60 min pi of ^68^Ga-NOTA-MG7. The tumor, liver and kidneys were immediately separated to perform frozen slice study by immunofluorescence staining. Briefly, the slice was first fixed and then washed three times with PBS and incubated with TRITC for 30 minutes. DAPI was used for nuclear staining.

### Statistical Analysis

Statistical analysis was performed using the statistical software package SPSS 13.0 (SPSS, Chicago, Illinois, USA), and quantitative data were expressed as the mean ± SD. All tests of significance were two-sided, and P values <0.05 were considered to be statistically significant.

## Author Contributions

B.X., J.P.Y. and X.W.L. completed the main experiment and wrote the first draft of the paper. C.L. and L.J.L. prepared figure 4. Z.Y.Q. prepared figure 5. L.P.Y. and Y.Z.N. analyzed the data. K.C.W. and J.W. designed the research. All authors have reviewed the final version of the manuscript and approve it for publication.
